# Prospective Evaluation of Quality‐of‐Life Improvement After Dapagliflozin Initiation in a Greek Population With HFrEF: The EVOLUTION‐HF 12‐Month Results

**DOI:** 10.1002/clc.70466

**Published:** 2026-09-06

**Authors:** Victoria Potoupni, Aristi Boulmpou, Spyridon Maragkoudakis, Alexandros Briasoulis, Emmanouil Foukarakis, Alexia Stavrati, Konstantinos Papadopoulos, Katerina K. Naka, Anna Kotsia, Ioannis Parissis, Antonios Ziakas, Stamatis Adamopoulos, Dimitrios Tziakas, Sotirios Patsilinakos, Melanie Konstantinidou, Periklis Davlouros, Zacharias Christogiannis, Konstantinos Tsitlakidis, Ioannis Pantelakis, Georgios Stefanakis, Marios Kolios, Ioannis Baltogiannis, Evangelos Voltyrakis, Christos Varounis, Andreas Samothrakitis, Dimitrios Krikidis, Panagiotis Tsialoukis, Zoe Paparepa, Dimitrios Gourlis, Nikolaos Papoutsidakis, Apostolos Karavidas, Christodoulos Papadopoulos

**Affiliations:** ^1^ Third Cardiology Department, Hippokrateion General Hospital, School of Medicine Aristotle University of Thessaloniki Thessaloniki Greece; ^2^ Department of Cardiology Chania General Hospital ‘Agios Georgios’ Chania Greece; ^3^ Department of Clinical Therapeutics, Alexandra Hospital, Faculty of Medicine National and Kapodistrian University of Athens Psachna Greece; ^4^ Department of Cardiology Venizeleion General Hospital Heraklion Greece; ^5^ Department of Cardiology General Hospital G. Papanikolaou Thessaloniki Greece; ^6^ 2nd Cardiology Department “Korgialenio‐Benakio” Red Cross Hospital Athens Greece; ^7^ 2nd Department of Cardiology, Faculty of Medicine School of Health Sciences, University of Ioannina, and University Hospital of Ioannina Ioannina Greece; ^8^ Department of Cardiology Ioannina State General Hospital “G. Chatzikosta” Ioannina Greece; ^9^ Emergency Medicine Department, Attikon University Hospital National and Kapodistrian University of Athens Athina Greece; ^10^ Department of Cardiology, AHEPA General University Hospital of Thessaloniki Aristotle University of Thessaloniki Thessaloniki Greece; ^11^ Heart Failure, Transplant and Mechanical Circulatory Support Units Onassis Cardiac Surgery Center Athens Greece; ^12^ Department of Cardiology, University Hospital of Alexandroupolis, Medical School Democritus University of Thrace Alexandroupolis Greece; ^13^ Department of Cardiology Konstantopouleio General Hospital Athens Greece; ^14^ Department of Cardiology Papageorgiou Hospital Thessaloniki Greece; ^15^ Department of Cardiology, Faculty of Medicine School of Health Sciences, University Hospital of Patras Rio Greece; ^16^ Cardiologist, Private Practice Greece; ^17^ 2nd Cardiology Department, Hippokrateion General Hospital, Medical School Aristotle University of Thessaloniki Thessaloniki Greece; ^18^ AstraZeneca Greece Marousi Greece; ^19^ Department of Cardiology “G. Gennimatas” General Hospital Athens Greece

**Keywords:** diabetes mellitus, heart failure, outcomes, quality of life, SGLT2 inhibitors

## Abstract

**Introduction:**

Heart failure with reduced ejection fraction (HFrEF), defined by a left ventricular ejection fraction ≤ 40%, remains a major global health challenge, associated with substantial morbidity, mortality, and impaired quality of life (QoL), particularly in patients with higher NYHA class. Sodium–glucose cotransporter‐2 (SGLT2) inhibitors have emerged as a cornerstone therapy for HFrEF, significantly reducing hospitalizations and mortality regardless of glycemic status, sex, race, or comorbidities.

**Methods:**

EVOLUTION‐HF was an observational, multi‐center, longitudinal cohort study involving 257 consecutive patients diagnosed with HFrEF who initiated dapagliflozin in a routine clinical setting in Greece. The primary objectives were to characterize baseline demographic and clinical features of patients newly initiated on dapagliflozin for HFrEF and to evaluate dapagliflozin treatment patterns, including discontinuation timing, reasons for discontinuation, and concomitant heart failure and glucose‐lowering therapies over time. The secondary objectives are to describe patient‐reported outcomes using the KCCQ‐23 and to assess adherence to dapagliflozin among patients with HFrEF.

**Results:**

A total of 257 patients were enrolled and the follow‐up period lasted for 12 months. Significant improvements among all KCCQ‐23 scores were observed from baseline to 12‐months post dapagliflozin initiation, revealing that the initiation and optimization of guideline‐directed medical therapy in routine clinical practice provides improvement to quality of life over time.

**Conclusions:**

Dapagliflozin was safe and well‐tolerated throughout the observation period. Although benefits are observed in most patients, individuals aged > 65 years, with prior myocardial infarction, or recent hospitalizations show a diminished response, underscoring the need for tailored management and closer follow‐up in these higher‐risk groups.

## Graphical abstract

Among patients with HFrEF, initiation of dapagliflozin resulted in significant improvements in quality of life, as demonstrated by increased KCCQ‐23 scores during follow‐up.

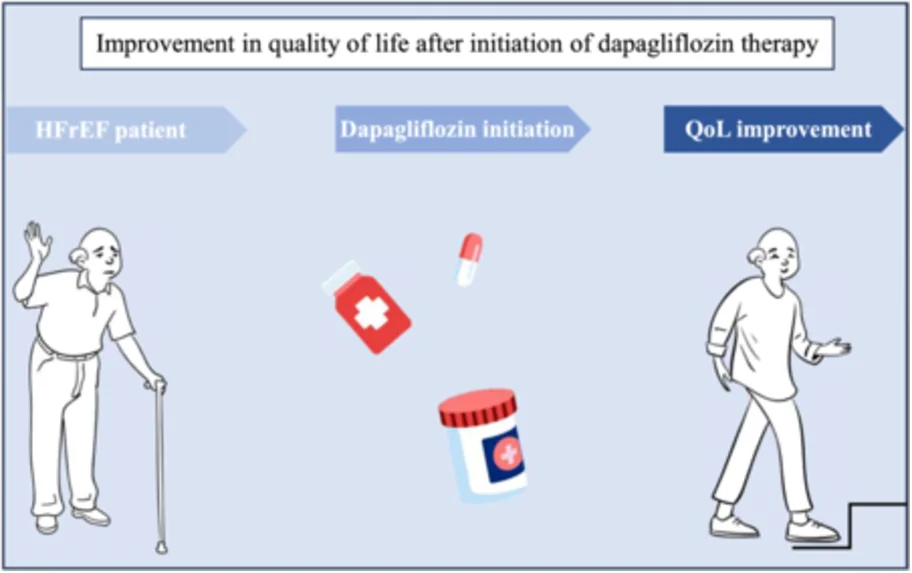

**Abbreviations**
AF: Atrial Fibrillation
BB: Beta‐Blocker
CAD: Coronary Artery Disease
CKD: Chronic Kidney Disease
COPD: Chronic Obstructive Pulmonary Disease
CRT: Cardiac Resynchronization Therapy
CSS: Clinical Summary Score
FAS: Full Analysis Set
HF: Heart Failure
HFrEF: Heart Failure with reduced Ejection Fraction
ICD: Implantable Cardioverter ‐ Defibrillator
IQR: Interquartile Range
KCCQ‐23: Kansas City Cardiomyopathy Questionnaire – 23
LVEF: Left Ventricular Ejection Fraction
MI: Myocardial Infarction
MRA: Mineralocorticoid Receptor Antagonist
NYHA: New York Heart Association
OSS: Overall Summary Score
PLS: Physical Limitation Score
PROs: Patient‐Reported Outcomes
QoL: Quality of Life
RAASi: Renin‐Angiotensin‐Aldosterone System inhibitors
SD: Standard Deviation
SGLT2: Sodium–Glucose Cotransporter‐2
T2DM: Type 2 Diabetes Mellitus
TSS: Total Symptom Score

## 1 Introduction

Heart failure (HF) is no longer viewed as a discrete pathological diagnosis, but rather as a complex clinical syndrome resulting from structural or functional impairments in ventricular filling or ejection. It is characterized by a constellation of symptoms, most notably dyspnea, fatigue, and exercise intolerance. These clinical manifestations are frequently accompanied by objective physical signs of congestion, such as elevated jugular venous pressure, pulmonary rales, and peripheral edema. Identification of the underlying etiology is essential for guiding therapeutic decisions; even though myocardial systolic or diastolic dysfunction are the most common causes, valvular disease, myopericardial syndromes, rhythm disorders, or conduction abnormalities, may also contribute to the development and progression of HF [1].

HF with reduced ejection fraction (HFrEF), characterized by a left ventricular ejection fraction (LVEF) of ≤ 40% [1], globally remains a major challenge for healthcare systems, associated with significant morbidity and mortality. Beyond its impact on survival, HFrEF profoundly impairs daily functioning and overall quality of life (QoL) of the affected individuals; higher New York Heart Association (NYHA) class has been demonstrated to strongly correlate with lower health‐related QoL [2].

In recent years, sodium‐glucose cotransporter‐2 (SGLT2) inhibitors have emerged as a cornerstone therapy for HF, offering significant reductions in hospitalization rates and overall mortality, independently of gender, race, glycemic status or major co‐ morbidities [3, 4, 5, 6]. The DAPA‐HF trial demonstrated that dapagliflozin significantly reduced cardiovascular death and HF‐related hospitalizations while improving symptoms and QoL in both diabetic and non‐diabetic patients with HFrEF [7]. Understanding whether improvements in symptoms are sustained, progressive, or variable across individuals is critical for optimizing patient counseling, setting expectations, and guiding therapeutic strategies.

While the landmark DAPA‐HF trial established the efficacy of dapagliflozin in controlled settings, the longitudinal trajectory of patient‐reported outcomes (PROs) in routine clinical practice warrants further investigation. Utilizing data from the EVOLUTION‐HF Greek cohort, this study examines the temporal dynamics of health‐related QoL following dapagliflozin initiation. Our findings provide critical real‐world evidence to bridge the gap between randomized controlled trials and the daily management of HFrEF.

## 2 Materials and Methods

EVOLUTION‐HF was an observational, multi‐center, longitudinal cohort study involving consecutive patients diagnosed with HFrEF who initiated dapagliflozin in a routine clinical setting. To capture early treatment effects while ensuring stable initiation, enrollment was restricted to patients between 14‐ and 60‐days post‐initiation. Notably, all therapeutic decisions, including dapagliflozin dosing and treatment duration, were made at the discretion of the treating physician according to local labeling, without protocol‐mandated interventions. Recruitment occurred across a diverse network of 16 hospital sites and 11 private practices between December 2022 to November 2023. The study concluded its 12‐month follow‐up period on December 3rd, 2024.

Patients were eligible for inclusion if they met all of the following criteria: (i) age ≥ 18 years, (ii) received dapagliflozin in the context of HFrEF (EF ≤ 40%) according to local labeling, and (iii) provided signed informed consent prior to enrollment. Patients were excluded if any of the following applied: (i) enrollment within less than 14 or more than 60 days after dapagliflozin initiation, (ii) prior treatment with dapagliflozin or any other SGLT2 inhibitor, (iii) initiation of dapagliflozin outside the approved local HF label, or (iv) a diagnosis of type 1 diabetes mellitus prior to enrollment.

Data were captured at enrollment and during subsequent follow‐up assessments at 3, 6, 9, and 12 months, synchronized with routine clinical visits. This comprehensive dataset comprised clinical parameters recorded by participating physicians and PROs. Specifically, health‐related QoL was evaluated using the Kansas City Cardiomyopathy Questionnaire (KCCQ‐23) [8], allowing for a longitudinal assessment of the patient's perspective alongside traditional clinical metrics. The KCCQ‐23 was utilized as a validated, disease‐specific instrument to quantify health status across several domains, primarily focusing on the Physical Limitation Score (PLS) and the Total Symptom Score (TSS), which accounts for symptom frequency and burden, as well as social limitation and QoL. Individual domains are aggregated into two primary composite measures: the Clinical Summary Score (CSS), which integrates the PLS and TTS to reflect a patient's functional and symptomatic status, and the Overall Summary Score (OSS), providing a global assessment of the patient's health status. All scores are transformed to a 0–100 scale, where higher scores represent superior health status and fewer functional limitations [8]. Clinically meaningful change for OSS and CSS was defined as a ≥ 5‐point increase from baseline at any time during the study observation period, whereas for TSS and PLS was defined as a ≥ 10‐point increase. These thresholds were applied post hoc, in line with the available literature [9, 10, 11].

Some follow‐up data were missing due to non‐attendance at scheduled visits. A complete case analysis was performed, including only participants with available data at each time point. No imputation was applied. For multivariable regression analyses, covariates with more than 10% missing data were excluded.

### 2.1 Statistical Analysis

Statistical analyses were conducted using SAS® software version 9.4 (SAS Institute Inc. Cary, NC, USA). Consistent with the observational design of the study, most analyses were descriptive.

Categorical variables are presented as frequencies (n, %) with 95% Clopper‐Pearson confidence intervals for binomial proportions, where applicable. Continuous variables are summarized using available observations (n), number of missing observations, mean, standard deviation (SD), median, 25th and 75th percentiles, and minimum and maximum values. The normality of continuous variables was assessed using the Shapiro‐Wilk test.

Univariable logistic regression analyses were conducted to assess associations between baseline characteristics and clinically meaningful improvement in the KCCQ‐23 OSS, CSS ‐ defined as a ≥ 5‐point increase from baseline at any time during the study observation period‐, TSS and PLS‐ defined as a ≥ 10‐point increase from baseline at any time during the observation period.

Multivariable logistic regression model included established prognostic factors in HFrEF: sex, smoking status, age at initial HF diagnosis (< 65 vs. ≥ 65 years), duration of HF at index date, NYHA functional class (Class II vs. III/IV), left ventricular ejection fraction (LVEF), hospitalization for HF within 12 months prior to index date, type 2 diabetes mellitus (T2DM), prior myocardial infarction (MI), atrial fibrillation (AF), implanted cardiac device therapy (ICD), and ongoing treatment with beta‐blockers (BB), mineralocorticoid receptor agonists (MRA), and renin‐angiotensin system inhibitors (RASi) at index date. A *p* value < 0.05 was considered statistically significant. For the initial step of multivariable modeling, candidate covariates were selected based on a combination of statistical significance, data completeness, and clinical relevance. Specifically, variables that were statistically significant in univariable analyses (*p* < 0.05), as well as those with *p* < 0.20, were considered. In cases where variables were assessed in both continuous and categorical forms, or using different categorization thresholds, the form with the lowest *p*‐value was selected. To ensure model stability and reliability, only variables with a missing data rate of less than 10% were included. Additionally, covariates deemed clinically important based on prior evidence were retained regardless of statistical significance. Covariates exhibiting significant imbalance between comparison groups were not prioritized for inclusion unless supported by clinical relevance.

## 3 Results

A total of 257 patients were consecutively included in the study. At enrollment, 250 patients (97.35%) were managed in the outpatient setting, while seven patients (2.7%) were hospitalized. HF was the primary reason for hospitalization in three patients, while the remaining four were admitted for other cardiovascular‐related indications. Baseline demographics and clinical characteristics of the study population are summarized in Table 1.

**TABLE 1:** Baseline demographics and clinical characteristics of total study cohort (*N* = 257).

Study population characteristics	Total population (*N* = 257)
Care setting on the date of enrollment	
Outpatient	250 (97.3)
Inpatient	7 (2.7)
Age at dapagliflozin initiation, years	67.9 ± 12.0
Sex	
Male	195 (75.9)
Female	62 (24.1)
Ethnic origin (Greek)	243 (94.6)
Race	
Caucasian	255 (99.2)
Asian	2 (0.8)
Smoking status	
Current smoker	45 (17.5)
Ex‐smoker	70 (27.2)
Never smoker	142 (55.3)
Body mass index, kg/m^2^	27.6 ± 4.7

*Note*: Categorical variables are presented as frequencies and percentages (*n*, %). Continuous variables are presented as mean ± standard deviation (SD). All data reflect patient status at the time of dapagliflozin initiation.

In the full analysis set (FAS), the median time from dapagliflozin initiation (index date) to study enrollment was 24.0 days (IQR, 17.0–24.0), reflecting enrollment early after treatment initiation.

### 3.1 HF Characteristics

The median age at initial HF diagnosis was 67.0 years. The principal causes of HF were non‐ischemic in 45.1%, ischemic in 41.6%, while the etiology was classified as unknown in 13.2% (Table 2). With respect to disease duration, 50.6% of patients had been diagnosed with HF within 3 months prior to the index date.

**TABLE 2:** HF characteristics, LVEF and ECG rhythm data of the study population.

HF characteristics	*N* (%)
Patient's age at initial HF diagnosis (years) (categorical)	
< 45 years	17 (6.6)
45 to < 55 years	35 (13.6)
55 to < 65 years	63 (24.5)
65 to < 75 years	77 (30.0)
75 to < 85 years	50 (19.5)
≥ 85 years	15 (5.8)
Patient's principal cause of HF	
Ischemic	107 (41.6)
Non‐ischemic	116 (45.1)
Unknown	34 (13.2)
HF duration (time from initial HF diagnosis to index date)	
≤ 3 months	130 (50.6)
> 3–6 months	10 (3.9)
> 6–12 months	13 (5.1)
> 1–2 years	17 (6.6)
> 2–5 years	35 (13.6)
> 5–10 years	33 (12.8)
> 10 years	19 (7.4)
NYHA functional class based on most recent assessment	
Class II	130 (57.3)
Class III	87 (38.3)
Class IV	10 (4.4)
LVEF (%) (categorical)	
15	4 (1.6)
> 15 to < 20	2 (0.8)
20 to < 30	46 (17.9)
30 to < 40	120 (46.7)
40	85 (33.1)
ECG	
Sinus rhythm	158 (75.2)
Non‐sinus rhythm	52 (24.8)
Hospitalization on index day	
Yes	67 (26.1)
No	190 (73.9)
Hospitalized for HF within the previous 12 months	32 (16.8)

Assessment of LVEF at enrollment was available for all patients in the FAS. The median (IQR) LVEF was 35.0% (30.0–40.0). Overall, 33.1% of patients had an LVEF of 40%, 46.7% had an LVEF between 30% and < 40%, and the remaining 20.2% had an LVEF of < 30%.

Among evaluable patients with available NYHA functional class assessment (*N* = 227), 57.3% were classified as class II, 38.3% as class III, and 4.4% as class IV (Table 2).

### 3.2 Clinically Significant Medical History and Concomitant Medication

A history of at least one clinically significant comorbidity or cardiovascular event prior to the index date was reported in 96.5% of patients. The most frequent documented conditions included hypertension, dyslipidemia, AF, prior MI, coronary revascularization, T2DM, implantable cardioverter‐defibrillator (ICD) implantation, chronic kidney disease (CKD), and chronic obstructive pulmonary disease (COPD) (Table 3).

**TABLE 3:** Most common medical conditions/events at baseline.

Medical History	*N* (%)
Presence of any clinically significant medical condition/event	
One medical condition/event	30 (11.7)
Two medical conditions/events	58 (22.6)
Three medical conditions/events	44 (17.1)
Four medical conditions/events	55 (21.4)
Five medical conditions/events	61 (23.7)
Cardiac disorders	187 (72.8)
Atrial fibrillation	88 (34.2)
Myocardial infraction	86 (33.5)
Coronary artery disease	20 (7.8)
Metabolism and nutrition disorders	138 (53.7)
Type 2 diabetes mellitus	73 (28.4)
Dyslipidemia	56 (21.8)
Surgical and medical procedures	121 (47.1)
Coronary revascularisation	77 (30.0)
ICD insertion	42 (16.3)
Vascular disorders	133 (51.8)
Hypertension	128 (48.8)
Renal and urinary disorders	40 (15.6)
CKD	40 (15.6)
Respiratory, thoracic and mediastinal disorders	22 (8.6)
Neoplasms benign, malignant and unspecified	17 (6.6)
Nervous system disorders	14 (5.4)
Psychiatric disorders	10 (3.9)

At baseline, HF‐directed pharmacotherapy was reported in 98.4% of patients, while treatments for other cardiorenal indications were reported in 81.3% and glucose‐lowering therapies in 23.0% of patients (Supporting Information S1: Table 1).

### 3.3 KCCQ‐23 Total and Domain Scores at Enrollment

At enrollment, the KCCQ‐23 questionnaire was completed by 250 patients. Among individual domains, symptom stability demonstrated the lowest median score [median (IQR): 50.0 (50.0–75.0)], indicating recent changes in symptoms, whereas higher median scores were observed for physical limitation, symptom frequency, symptom burden, and social limitation (Supporting Information S1: Table 2).

Median (IQR) summary scores at enrollment were 83.3 (72.9–95.8) for the TSS, 82.3 (66.7–91.7) for the CSS, and 79.2 (59.9–88.0) for the OSS. Based on these summary measures, the majority of patients were classified as having good or excellent health status at baseline (Table 4).

**TABLE 4:** Health status based on KCCQ‐23 summary scores at enrollment and at 12‐months.

	Baseline			Follow up		
Health status based on KCCQ‐23 summary scores	N (%)	95% Clopper‐Pearson CI		N (%)	95% Clopper‐Pearson CI	
		Lower limit	Upper limit		Lower limit	Upper limit
Total symptom score (TSS) status	**(*N* = 250)**			**(*N* = 208)**		
Poor (score < 25)	7 (2.8)	1.13	5.68	2 (1.0)	0.12	3.43
Fair (25 ≤ score < 50)	14 (5.6)	3.10	9.22	3 (1.4)	0.30	4.16
Good (50 ≤ score < 75)	50 (20.0)	15.22	25.50	17 (8.2)	4.83	12.76
Excellent (75 ≤ score ≤ 100)	179 (71.6)	65.58	77.10	186 (89.4)	84.42	93.25
Clinical summary score (CSS) status	**(*N* = 243)**			**(*N* = 201)**		
Poor (score < 25)	7 (2.9)	1.17	5.84	0		
Fair (25 ≤ score < 50)	20 (8.2)	5.10	12.43	6 (3.0)	1.10	6.38
Good (50 ≤ score < 75)	56 (23.0)	17.90	28.86	26 (12.9)	8.63	18.38
Excellent (75 ≤ score ≤ 100)	160 (65.8)	59.51	71.79	169 (84.1)	78.27	88.85
Overall summary score (CSS) status	**(*N* = 230)**			**(*N* = 189)**		
Poor (score < 25)	11 (4.8)	2.41	8.40	2 (1.1)	0.13	3.77
Fair (25 ≤ score < 50)	29 (12.6)	8.61	17.60	6 (3.2)	1.17	6.78
Good (50 ≤ score < 75)	62 (27.0)	21.34	33.18	22 (11.6)	7.44	17.09
Excellent (75 ≤ score ≤ 100)	128 (55.7)	48.98	62.18	159 (84.1)	78.12	89.03

The proportion of patients with baseline KCCQ‐23 scores that allowed the assessment of clinically meaningful change at follow‐up varied across domains and summary scores and is detailed in Supporting Information S1: Table 3.

### 3.4 Medication at 12 Months

**FIGURE 1:**
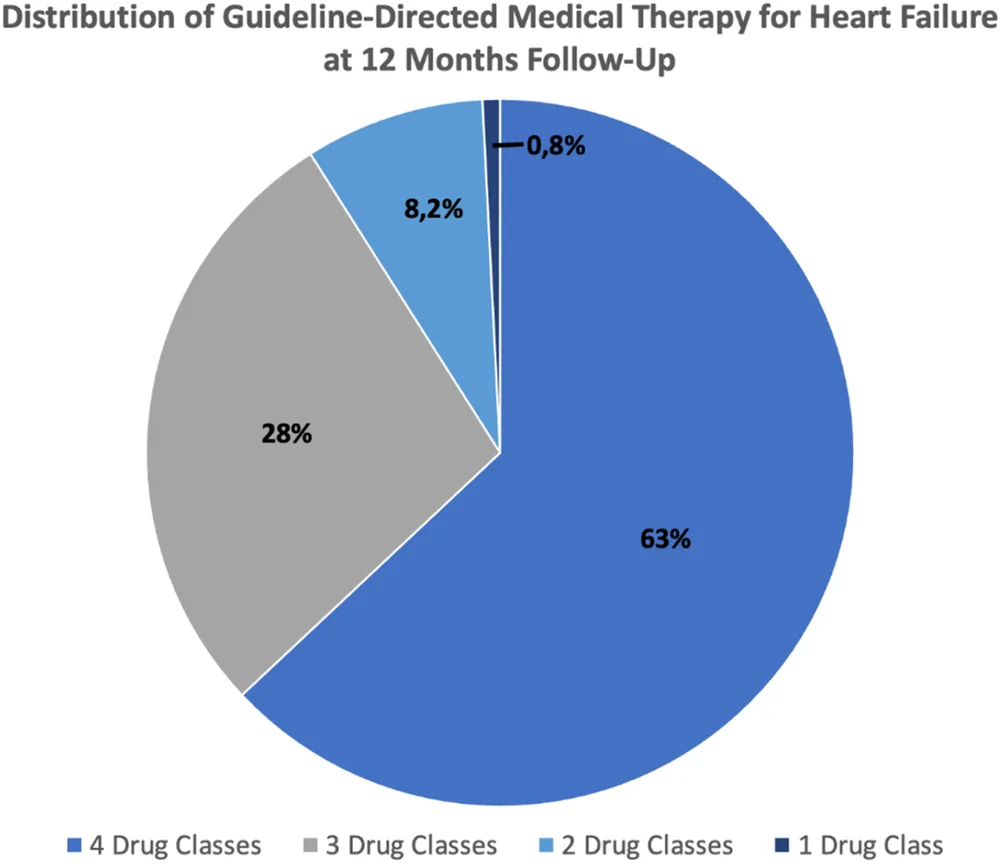
Treatment patterns for HFrEF at 12 months in a routine clinical setting. Percentages represent patients treated with 4, 3, 2, or 1 disease‐modifying drug classes (including SGLT2 inhibitors, beta‐blockers, MRAs, and RAAS inhibitors). Data reflects high adherence to the four‐pillar therapy approach within the EVOLUTION‐HF study population.

During the 12‐month follow‐up period, permanent discontinuation of dapagliflozin during was reported in six patients. The reason for discontinuation was adverse drug reaction in four patients (euglycemic diabetic ketoacidosis in two patients, fungal genital infection in one patient, dysuria in one patient) and physician decision in two patients. At the 12‐month study visit, the majority of patients were receiving comprehensive guideline‐directed medical therapy for HF. Specifically, 63.0% of patients were treated with all four disease‐modifying drug classes (SGLT2 inhibitors, BBs, MRAs, and RAASi), 28.0% were receiving three classes, 8.2% were receiving two classes, and 0.8% were receiving only one disease‐modifying drug class (Figure 1, Supporting Information S1: Table 4).

### 3.5 KCCQ‐23 Total and Domain Scores at 12 Months Post‐Index

**FIGURE 2:**
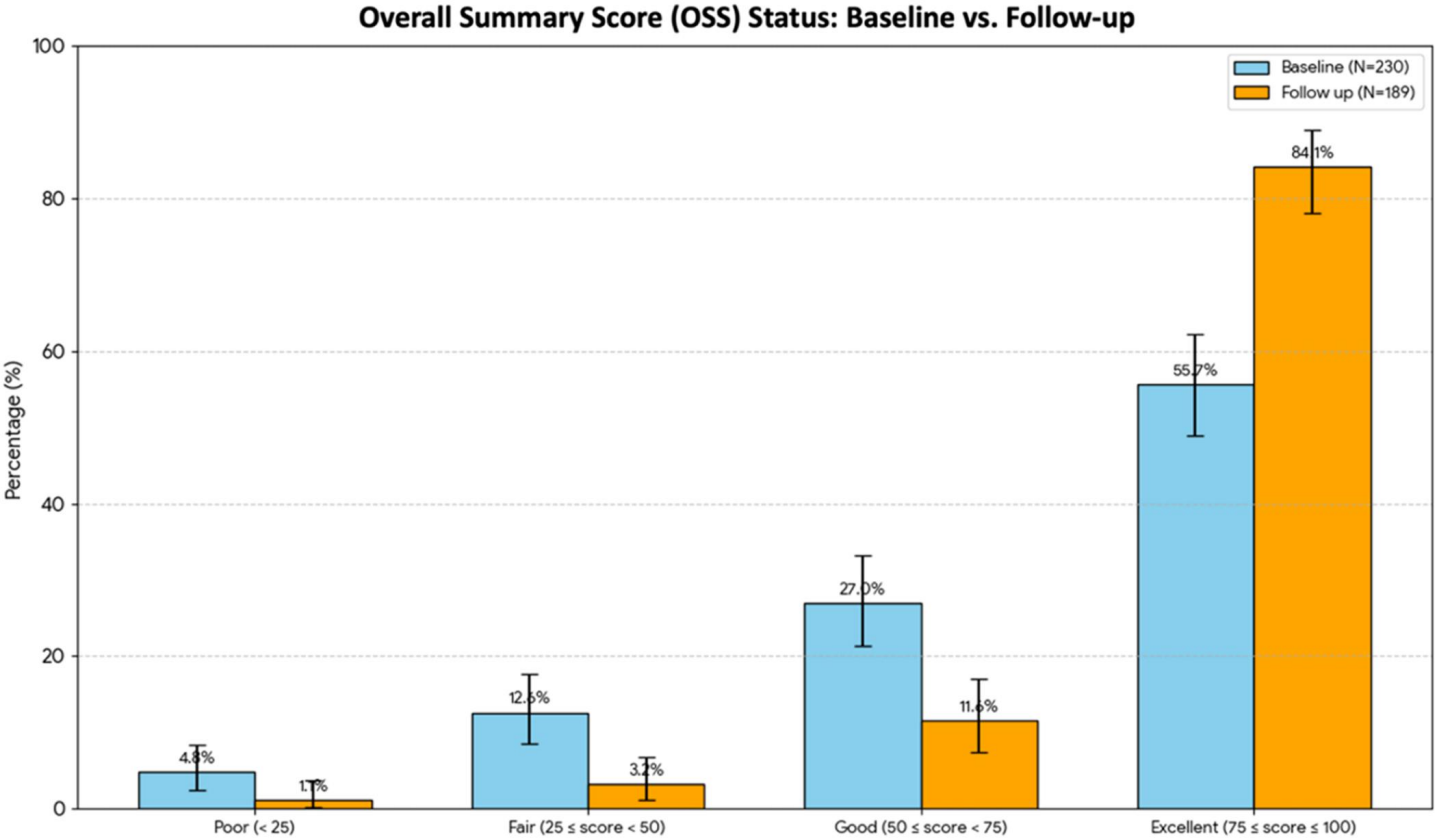
Distribution of KCCQ‐23 Overall Summary Score (OSS) status at baseline and follow‐up. The chart illustrates a significant health status improvement, characterized by a substantial increase in patients achieving “Excellent” scores and a corresponding decrease across “Poor,” “Fair,” and “Good” categories at the follow‐up stage.

At the 12‐month follow‐up visit, the KCCQ‐23 questionnaire was completed by 209 of the 245 participants attending the visit. Among individual domains, symptom stability remained the lowest‐scoring domain [median (IQR): 50.0 (50.0–75.0)], while substantial improvements were observed across QoL, physical limitation, symptom frequency, symptom burden, and social limitation, indicating improved health status in most domains (Figure 2, Supporting Information S1: Table 5).

Median (IQR) summary scores at 12 months were 94.8 (85.4–100.0) for the TSS, 93.8 (84.9–100.0) for the CSS, and 91.7 (83.5–99.0) for the OSS. Based on the TSS, 89.4% of patients were classified as having excellent health status, with similarly high proportions classified as excellent according to CSS (84.1%) and OSS (84.1%).

Among patients with paired baseline and 12‐month KCCQ‐23 data, mean changes from enrollment to 12 months demonstrated clinically significant improvements across all summary scores and key domains. Mean (95% CI) changes were +11.8 (8.7–14.9) for the PLS, +9.6 (6.7–12.5) for the TSS, +11.1 (8.4–13.9) for the CSS, and +14.2 (11.0–17.3) for the OSS (Supporting Information S1: Table 5).

Using pre‐specified threshold for clinically meaningful improvement, 59.1% of patients achieved a ≥ 5‐point increase in OSS and 54.4% achieved a ≥ 5‐point increase in CSS from baseline to 12 months. Improvements of ≥ 10 points were observed in 39.3% of patients for TSS and 40.0% for PLS among those with paired data (Supporting Information S1: Table 5).

**FIGURE 3:**
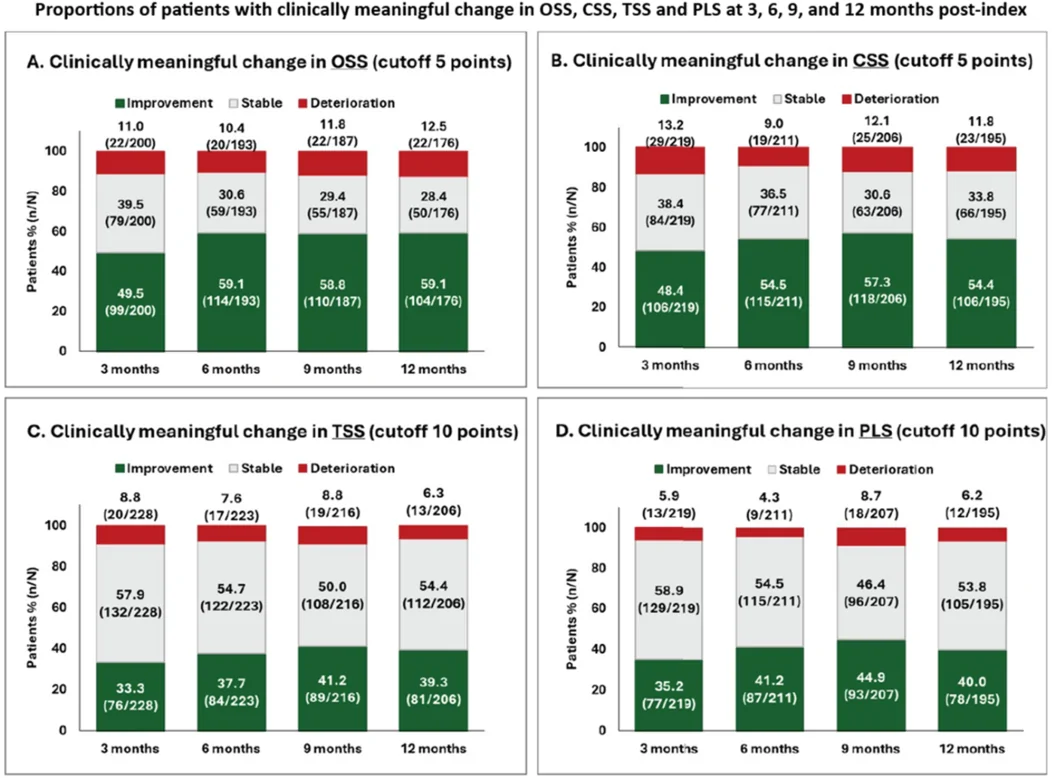
Proportions of patients with clinically meaningful change in OSS, CSS, TSS and PLS at 3, 6, 9, and 12 months post‐index.

When changes across all post‐enrollment visits were considered, clinically relevant improvement at any time during follow‐up was observed in 73.2% of patients for OSS, 68.8% for CSS, 53.5% for TSS, and 53.4% for PLS (Figure 3).

### 3.6 Association Between KCCQ‐23 Scores Improvement and Baseline Characteristics

**FIGURE 4:**
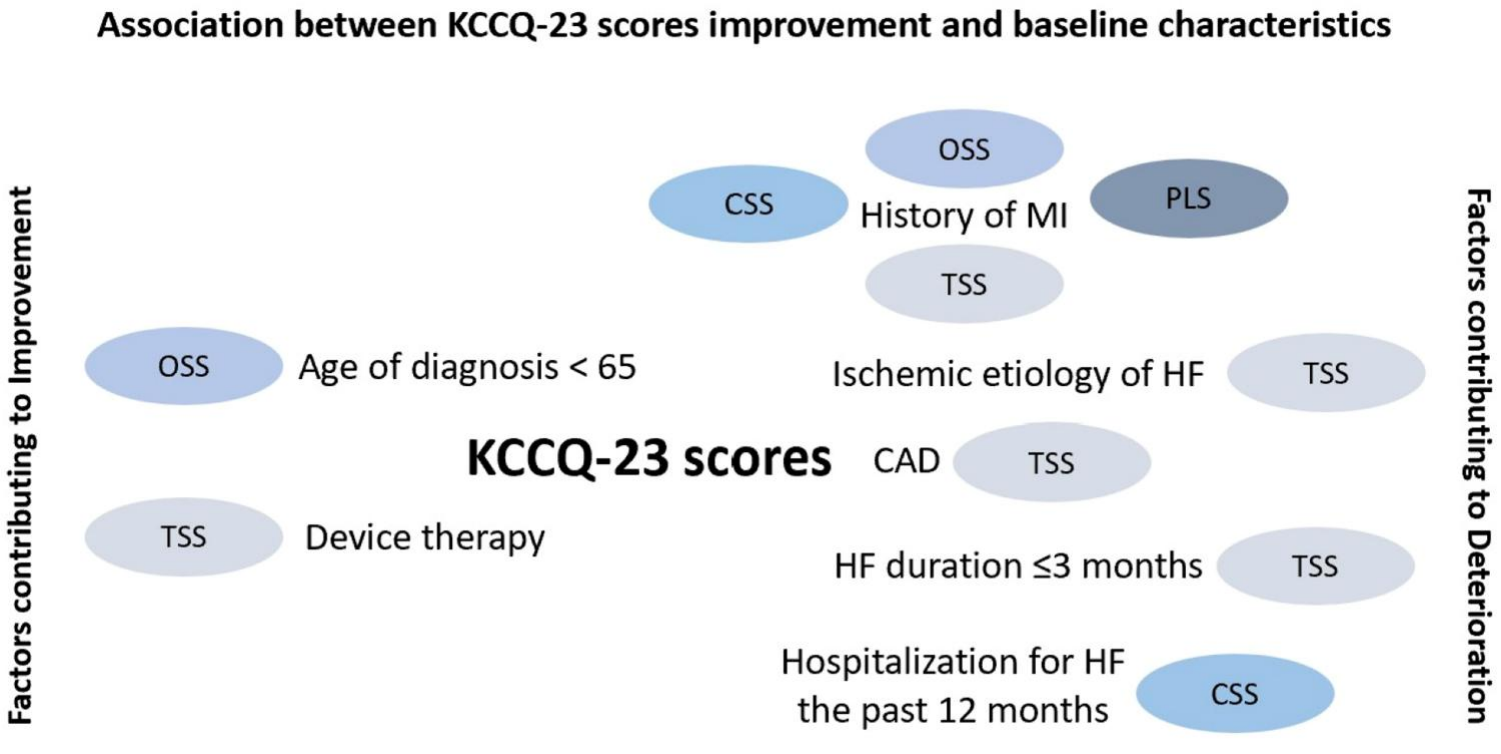
Association between KCCQ‐23 scores improvement and baseline characteristics.

In the univariable analysis, age at HF diagnosis < 65 years was associated with higher odds of achieving a clinically meaningful improvement in KCCQ‐23 OSS, defined as an increase in ≥ 5 points from baseline (OR 2.01, 95% CI 1.02–3.95). A history of MI was associated with lower odds of achieving clinically meaningful improvement in the KCCQ‐23 OSS (OR 0.51, 95% CI 0.26–0.97) and CSS (OR 0.49, 95% CI 0.27–0.89), defined as an increase of ≥ 5 points from baseline, as well as with KCCQ‐23 PLS improvement, defined as an increase of ≥ 10 points from baseline (OR 0.46, 95% CI 0.25–0.83). Clinically meaningful improvement in the KCCQ‐23 TSS, defined as an increase of ≥ 10 points from baseline, was associated with the principal cause of HF, HF duration, presence of coronary artery disease (CAD), prior MI, and use of device therapy (ICD and/or CRT). Specifically, ischemic etiology of HF, CAD, prior MI, and HF duration ≤ 3 months were associated with lower odds of improvement, whereas use of device therapy was associated with higher odds of improvement (Supporting Information S1: Table 6, Figure 4).

In the multivariable logistic regression model, prior MI remained independently associated with a lower likelihood of clinically meaningful OSS (OR 0.45, 95% CI 0.23–0.89), CSS (OR 0.37, 95% CI 0.18–0.75), PLS (OR 0.47, 95% CI 0.25–0.85) and TSS (OR 0.36, 95% CI 0.20–0.76) improvement. Hospitalization for HF within the 12 months prior to the index date was associated with lower odds of clinically meaningful CSS improvement (OR 0.47, 95% CI 0.23–0.95). Use of device therapy was associated with higher odds of KCCQ‐23 TSS improvement (OR 3.01, 95% CI 1.45–6.24) (Supporting Information S1: Table 6, Figure 4).

## 4 Discussion

The KCCQ‐23 questionnaire is a validated, disease‐specific instrument designed to assess health status in patients with HF, covering symptoms, physical and social limitations, and QoL. It consists of 23 items summarized into distinct domain scores (PLS and TSS), as well as two primary composite measures: the CSS and the OSS [8].

Each score is transformed into a 0–100 scale, where higher values indicate superior health status and fewer symptoms. Changes of 5, 10, and 20 points are recognized as small, moderate, and large clinically meaningful shifts, respectively [8]. Recent meta‐analyses confirm that lower baseline KCCQ‐23 scores are potent predictors of adverse outcomes in HF; specifically, each 5‐point decrease is linked to a significant rise in all‐cause mortality and HF‐related hospitalizations [12].

In our cohort, prior MI emerged as a significant predictor of poor response, associated with a lower likelihood of improvement in OSS, CSS, TSS and PLS in patients with HFrEF receiving dapagliflozin. Furthermore, hospitalization for HF within the preceding 12 months was associated with reduced odds of achieving a clinically meaningful improvement in CSS, whereas the use of device therapy was significantly associated with TSS improvement. These findings highlight that recent clinical instability or specific therapeutic interventions are critical determinants of how a patient's health status evolves under SGLT2 inhibitor therapy.

Despite these, it should be mentioned that a notable proportion of patients had good or excellent KCCQ‐23 scores at baseline, which may have introduced ceiling effects. The proportion of patients with KCCQ‐23 scores at enrollment precluding the ability to demonstrate clinically meaningful change due to high baseline scores were 30.0% (70/243) for PLS, 32.8% (82/250) for TSS, 16.5% (40/243) for CSS and 10.0% (23/230) for OSS. To better contextualize the observed changes, baseline‐stratified data are shown at Supporting Information S1: Table 8.

Our findings align with data from major clinical trials. The DAPA‐HF study included 4744 patients with HFrEF treated with either dapagliflozin or placebo [7]. From baseline to 8 months, patients treated with dapagliflozin experienced a greater improvement in the KCCQ‐23 TSS compared with those receiving placebo. A higher proportion of patients in the dapagliflozin group achieved a clinically meaningful improvement of at least five points in the total score (58.3% vs. 50.9%; OR 1.15; 95% CI, 1.08–1.23), while a smaller proportion experienced a clinically significant worsening (25.3% vs. 32.9%; OR 0.84; 95% CI, 0.78–0.90; *p* < 0.001 for both comparisons).

In the same line, the DEFINE‐HF study randomized 263 patients with HFrEF to receive dapagliflozin or placebo. Compared with placebo, dapagliflozin was associated with a higher proportion of patients achieving a clinically meaningful improvement in KCCQ‐23 OSS at both 6 weeks (47% vs. 36%) and 12 weeks (43% vs. 33%). Similar findings were observed for the KCCQ‐23 CSS, with greater improvements in symptoms and physical limitations over time. Significant improvements were seen in most KCCQ‐23 domains (total symptoms, physical limitations, and quality of life), while social limitations did not differ between groups [13].

The DETERMINE program consisted of double‐blind, placebo‐controlled, multicenter trials evaluating the effects of dapagliflozin on patient‐reported symptoms, physical limitations, and exercise capacity. A total of 313 patients with HFrEF (DETERMINE‐Reduced) and 504 patients with HF with preserved ejection fraction (HFpEF) (DETERMINE‐Preserved) were enrolled. In patients with HFrEF, treatment with dapagliflozin for 16 weeks led to a statistically significant improvement in symptom burden as measured by the KCCQ‐23 TSS compared with placebo (*p* = 0.022). The PLS improvement did not reach conventional statistical significance. In patients with HFpEF, dapagliflozin did not produce statistically significant improvements in either KCCQ‐23 TSS or KCCQ‐23 PLS at 16 weeks compared with placebo [14].

Collectively, dapagliflozin has demonstrated efficacy and safety across the HF spectrum. In HFrEF, it reduces the risk of hospitalization and cardiovascular death while enhancing patient‐reported outcomes [6, 7]. In HFpEF, despite the mixed results in DETERMINE, other data suggest association with improved symptom scores, physical function, and health‐related QoL, supporting broad applicability regardless of diabetes status [13, 14, 15]. Importantly, initiation of dapagliflozin, whether in chronic stable HF or early during acute decompensation, is well tolerated, with no clinically meaningful increase in adverse events such as hypotension, acute kidney injury, or hypoglycemia [16]. These data support the early integration of dapagliflozin into guideline‐directed medical therapy to optimize both clinical outcomes and QoL.

Regarding the safety profile of dapagliflozin, treatment discontinuation due to adverse events was low and comparable to placebo across trials. In the DETERMINE‐Reduced study, nine patients (5.8%) in the dapagliflozin group discontinued treatment because of adverse events, compared with 7.6% in the placebo group [14]. In the DEFINE‐HF trial, 11 of 131 patients (8.4%) receiving dapagliflozin discontinued therapy due to adverse events, versus 12 of 132 patients (9.0%) in the placebo group [13]. Similarly, in the DAPA‐HF trial, treatment discontinuation due to adverse events occurred in 111 of 2368 patients (4.7%) in the dapagliflozin group and 116 of 2368 patients (4.9%) in the placebo group [7]. Taken together, these findings demonstrate a consistent and favorable tolerability profile for dapagliflozin, with no signal of excess treatment‐limiting adverse events compared with placebo across a broad spectrum of patients with HF.

Our study has several limitations warranting consideration. An important limitation of this study is its observational, single‐arm design without a comparator group, which precludes causal inference. Although improvements in health‐related QoL, symptom burden, and physical function were observed over 12 months, these changes cannot be attributed to dapagliflozin alone. Rather, they are likely associated with the concomitant use and optimization of guideline‐directed medical therapy in routine clinical practice. Second, the trial was conducted exclusively within a Greek population, which may restrict the generalizability of findings to other ethnic or geographic groups. Third, our participants were relatively healthy at baseline, with 71.6%, 65.8%, and 55.7% classified as having excellent health status by the TSS, CSS, and OSS, respectively, which may have limited the potential for further clinically meaningful improvement due to the already high baseline scores. Another limitation is that early treatment effects may have already occurred before the time of assessment, while patients who discontinued treatment early may not have been captured in the analysis, potentially introducing selection bias. Additionally, while the 12‐month follow‐up provided medium‐term data, longer‐term durability remains unknown. Finally, although the sample size was sufficient for primary endpoints, it may have limited statistical power for detecting subtle differences in subgroup analyses or rare adverse events.

In our study, prior myocardial infarction emerged as a distinct predictor of response, associated with a lower likelihood of QoL improvement across KCCQ‐23 domains. Similarly, patients diagnosed after the age of 65 or those with HF hospitalizations in the preceding 12 months were less likely to experience significant gains in health status. Accordingly, our findings suggest that, although the broader population may benefit from therapy, certain higher‐risk subgroups may experience less pronounced improvements in functional status. These patients may require closer monitoring, more frequent follow‐up, and potentially individualized therapeutic strategies to optimize functional recovery. Nevertheless, these observations should be interpreted with caution. Formal interaction testing was not performed, and residual confounding may be present, limiting the ability to draw definitive conclusions.

## 5 Conclusions

Dapagliflozin was generally well tolerated in our study, although a comprehensive assessment and comparison of adverse events was beyond the scope of the present analysis. The concomitant use of dapagliflozin with guideline‐directed medical therapy is associated with improvements in health‐related QoL, symptom burden, and physical function over 12 months in routine clinical practice. While these benefits extend to the majority of patients with HF, those with a history of myocardial infarction, age > 65 years, or recent hospitalizations exhibited an attenuated response. These findings suggest that dapagliflozin, when used as part of guideline‐directed medical therapy, may be associated with beneficial changes in patient‐reported outcomes and physical function. However, given the observational, single‐arm design, these findings should be interpreted as associative rather than as evidence of a direct treatment effect. Higher‐risk patients may warrant closer monitoring and individualized management.

## Author Contributions

Study conception and design: Z.P., D.G., N.P. Study coordination and supervision: C.P., A.Ka. Patient recruitment and data acquisition: V.P., S.M., A.Br., E.F., A.St., K.P., Κ.Κ.N., A.Ko., I.Par., A.Z., S.A., D.T., S.P., M.Kon., Z.C., K.T., I.Pan., G.S., M.Kol., I.B., E.V., C.V., A.Sa., D.K., P.T., A.Ka., C.P. Data curation and validation: V.P., N.P., C.P., A.Ka., Z.P., D.G. Statistical analysis: N.P., C.P., V.P., A.Bo. Interpretation of data: all authors. Manuscript drafting: V.P., A.Bo., C.P. Critical revision of the manuscript for important intellectual content: all authors. All authors reviewed and approved the final manuscript and agree to be accountable for all aspects of the work.

## Ethics Statement

EVOLUTION‐HF is a non‐interventional, prospective, longitudinal cohort study assessed by corresponding ethic committee. The investigation conforms with the principles outlined in the Declaration of Helsinki.

## Supporting information


**Table S1:** Concomitant HF, cardiorenal and metabolism modifying medications at dapagliflozin initiation and at 12‐months. **Table S2:** Health status based on KCCQ‐23 domain scores at enrolment. **Table S3:** Proportion of patients with KCCQ scores at enrolment precluding the ability to demonstrate clinically meaningful change at post‐enrolment visits. **Table S4:** Distribution of patients per number of disease‐modifying drug classes for HF received at baseline and at the 12‐months visit. **Table S5:** Proportion of patients with clinically relevant change in KCCQ‐23 health status from enrolment at 12 months post‐index among patients with paired enrolment and 12‐month data. **Table S6:** Association Between KCCQ‐23 Improvement and Baseline Characteristics. **Table S7:** Multivariable logistic regression analysis for the association of factors of interest with clinically relevant improvement in KCCQ‐23 Scores from enrolment at any time over sthe study observation period. **Table S8:** Proportion of patients with clinically relevant change in KCCQ‐23 health status from enrolment at 12 months post‐index among patients with paired enrolment and 12‐month data.

## Data Availability

The data that support the findings of this study are available from the corresponding author upon reasonable request. The data underlying this study are not publicly available due to ethical and privacy restrictions but may be made available from the corresponding author upon reasonable request and subject to approval by the relevant ethics committee.
