# Supplementary material for: Prospective Evaluation of Quality‐of‐Life Improvement After Dapagliflozin Initiation in a Greek Population With HFrEF: The EVOLUTION‐HF 12‐Month Results
Source: Clin Cardiol. 2026 Sep 6;49(9):e70466. doi: 10.1002/clc.70466 (PMC13546617; doi:10.1002/clc.70466)
Supplement: Supplementary file 1 [file CLC-49-e70466-s001.docx]

| **Drug classes** | **n (%) – Baseline**  **N = 257** | **n (%) – 12 months**  **N = 245** |
| --- | --- | --- |
| SGLT2i | 0 | 241 (98.4) |
| Beta-blockers | 233 (90.7) | 225 (91.8) |
| Antithrombotics (antiplatelets & anticoagulants) | 200 (77.8) | 187 (76.3) |
| Mineralocorticoid receptor antagonists | 200 (77.8) | 202 (82.4) |
| Diuretics | 173 (67.3) | 168 (68.6) |
| Angiotensin receptor-neprilysin inhibitors (ARNI) | 91 (35.4) | 110 (44.9) |
| ACE inhibitors (ACEi) | 61 (23.7) | 54 (22.0) |
| Angiotensin receptor blockers (ARB) | 50 (19.5) | 41 (16.7) |
| Antiarrhythmics | 20 (7.8) | 18 (7.3) |
| Calcium channel blockers (CCB) | 13 (5.1) | 13 (5.3) |
| If-channel inhibitor | 13 (5.1) | 14 (5.7) |
| Antihyperuricemic agents | 9 (3.5) | 10 (4.1) |
| Digoxin/Digitoxin | 7 (2.7) | 3 (1.2) |
| Anti-anginal medication | 5 (1.9) | 4 (1.6) |
| Organic nitrates | 4 (1.6) | 4 (1.6) |
| Other anticoagulants (XIa factor inhibitor) or placebo | 2 (0.8) | 1 (0.4) |
| Metformin | 36 (14.0) | 37 (15.1) |
| Insulin | 23 (8.9) | 19 (7.8) |
| DPP-4i | 20 (7.8) | 18 (7.3) |
| GLP-1-RA | 8 (3.1) | 9 (3.7) |
| Sulphonylurea | 1 (0.4) | 2 (0.8) |
| Lipid modifying agents | 90 (35.0) | 92 (37.6) |
| sGC stimulator | 0 | 4 (1.6) |

**Supplementary Table 1**: Concomitant HF, cardiorenal and metabolism modifying medications at dapagliflozin initiation and at 12-months

| **Health status based on KCCQ-23 domain scores** | **N (%)** | **95% Clopper-Pearson CI** | |
| --- | --- | --- | --- |
|  |  | **Lower limit** | **Upper limit** |
| **Physical limitation status (N=243)** | | | |
| Poor (score < 25) | 13 (5.3) | 2.88 | 8.97 |
| Fair (25 ≤ score < 50) | 27 (11.1) | 7.45 | 15.75 |
| Good (50 ≤ score < 75) | 48 (19.8) | 14.94 | 25.32 |
| Excellent (75 ≤ score ≤ 100) | 155 (63.8) | 57.40 | 69.83 |
| **Symptom stability status (N=250)** | | | |
| Poor (score < 25) | 8 (3.2) | 1.39 | 6.21 |
| Fair (25 ≤ score < 50) | 9 (3.6) | 3.70 | 6.72 |
| Good (50 ≤ score < 75) | 117 (46.8) | 40.49 | 53.19 |
| Excellent (75 ≤ score ≤ 100) | 116 (46.4) | 40.09 | 52.79 |
| **Symptom frequency status (N=250)** | | | |
| Poor (score < 25) | 8 (3.2) | 1.39 | 6.21 |
| Fair (25 ≤ score < 50) | 16 (6.4) | 3.70 | 10.19 |
| Good (50 ≤ score < 75) | 43 (17.2) | 12.74 | 22.46 |
| Excellent (75 ≤ score ≤ 100) | 183 (73.2) | 67.25 | 78.59 |
| **Symptom burden status (N=250)** | | | |
| Poor (score < 25) | 4 (1.6) | 0.44 | 4.05 |
| Fair (25 ≤ score < 50) | 17 (6.8) | 4.01 | 10.66 |
| Good (50 ≤ score < 75) | 39 (15.6) | 11.33 | 20.70 |
| Excellent (75 ≤ score ≤ 100) | 190 (76.0) | 70.21 | 81.16 |
| **Self-efficacy status (N=250)** | | | |
| Poor (score < 25) | 7 (2.8) | 1.13 | 5.68 |
| Fair (25 ≤ score < 50) | 31 (12.4) | 8.58 | 17.14 |
| Good (50 ≤ score < 75) | 71 (28.4) | 22.90 | 34.42 |
| Excellent (75 ≤ score ≤ 100) | 141 (56.4) | 50.01 | 62.64 |
| **Quality of Life status (N=249)** | | | |
| Poor (score < 25) | 24 (9.6) | 6.27 | 14.00 |
| Fair (25 ≤ score < 50) | 36 (14.5) | 10.34 | 19.45 |
| Good (50 ≤ score < 75) | 72 (28.9) | 23.37 | 34.98 |
| Excellent (75 ≤ score ≤ 100) | 117 (47.0) | 40.66 | 53.39 |
| **Social limitation status (N=233)** | | | |
| Poor (score < 25) | 23 (9.9) | 6.36 | 14.44 |
| Fair (25 ≤ score < 50) | 22 (9.4) | 6.01 | 13.95 |
| Good (50 ≤ score < 75) | 44 (18.9) | 14.07 | 24.51 |
| Excellent (75 ≤ score ≤ 100) | 144 (61.8) | 55.23 | 66.07 |

**Supplementary Table 2:** Health status based on KCCQ-23 domain scores at enrolment

|  | N (%) |
| --- | --- |
| **Physical limitation status (N=243)** |  |
| PLS at enrolment ≥10 to ≤90 allowing clinically meaningful change | 166 (68.3) |
| **PLS at enrolment <10 or >90 precluding clinically meaningful change** | **77 (31.7)** |
| <10 points precluding deterioration | 4 (1.6) |
| >90 points precluding improvement | 73 (30.0) |
| **Total symptom score (TSS) status (N=250)** |  |
| TSS at enrolment ≥10 to ≤90 allowing clinically meaningful change | 166 (66.4) |
| **TSS at enrolment <10 or >90 precluding clinically meaningful change** | **84 (33.6)** |
| <10 points precluding deterioration | 2 (0.8) |
| >90 points precluding improvement | 82 (32.8) |
| **Clinical summary score (CSS) status (N=243)** |  |
| CSS at enrolment ≥5 to ≤95 allowing clinically meaningful change | 203 (83.5) |
| **CSS at enrolment <5 or >95 precluding clinically meaningful change** | **40 (16.5)** |
| >95 points precluding improvement | 40 (16.5) |
| **Overall summary score (OSS) status (N=230)** |  |
| OSS at enrolment ≥5 to ≤95 allowing clinically meaningful change | 207 (90.0) |
| **OSS at enrolment <5 or >95 precluding clinically meaningful change** | **23 (10.0)** |
| >95 points precluding improvement | 23 (10.0 %) |

**Supplementary Table 3:** Proportion of patients with KCCQ scores at enrolment precluding the ability to demonstrate clinically meaningful change at post-enrolment visits.

| **Number of disease-modifying drug classes** | **n (%) – Baseline**  **N = 257** | **n (%) – 12 months**  **N = 245** |
| --- | --- | --- |
| **1 (one drug class)** | **2 (0.8)** | **1 (0.4)** |
| SGLT2i | 2 (0.8) | 0 |
| RAASi | 0 | 1 (0.4) |
| **2 (two drug classes)** | **30 (11.7)** | **19 (7.8)** |
| BB & SGLT2i | 23 (8.9) | 12 (4.9) |
| MRA & SGLT2i | 4 (1.6) | 3 (1.2) |
| RAASi & SGLT2i | 3 (1.2) | 3 (1.2) |
| BB & MRA | 0 | 1 (0.4) |
| **3 (three drug classes)** | **72 (28.0)** | **67 (27.3)** |
| BB & RAASi & SGLT2i | 29 (11.3) | 27 (11.0) |
| BB & MRA & SGLT2i | 28 (10.9) | 25 (10.2) |
| MRA & RAASi & SGLT2i | 15 (5.8) | 13 (5.3) |
| BB & MRA & RAASi | 0 | 2 (0.8) |
| **4 (four drug classes)** | **153 (59.5)** | **158 (64.5)** |
| BB & MRA & RAASi & SGLT2i | 153 (59.5) | 158 (64.5) |

**Supplementary Table 4**: Distribution of patients per number of disease-modifying drug classes for HF received at baseline and at the 12-months visit

|  |  | **95% Clopper-Pearson CI** | |
| --- | --- | --- | --- |
|  | **N (%)** | **Lower limit** | **Upper limit** |
| **Overall summary score (OSS) (N=176)** |  |  |  |
| **Improvement (increase of ≥5 points as compared to enrolment)** | 104 (59.1) | 51.44 | 66.43 |
| Deterioration (decrease of ≥5 points as compared to enrolment) | 22 (12.5) | 8.00 | 18.31 |
| Stable (change of <5 points in either direction compared to enrolment) | 50 (28.4) | 21.88 | 35.68 |
| Increase <5 points  *Baseline KCCQ-OSS score >95 points at enrolment* | 32 (18.2)  *5 (15.6)* | 12.78  *0.93* | 24.69  *6.50* |
| Decrease <5 points | 12 (6.8) | 3.57 | 11.61 |
| No change (score remains the same as at enrolment)  *Baseline KCCQ-OSS score >95 points at enrolment* | 6 (3.4)  *5 (83.3)* | 1.26  *0.93* | 7.27  *6.50* |
| **Clinical summary score (CSS) (N=195)** |  |  |  |
| **Improvement (increase of ≥5 points as compared to enrolment)** | 106 (54.4) | 47.09 | 61.49 |
| Deterioration (decrease of ≥5 points as compared to enrolment) | 23 (11.8) | 7.63 | 17.17 |
| Stable (change of <5 points in either direction compared to enrolment) | 66 (33.8) | 27.24 | 40.95 |
| Increase <5 points  *Baseline KCCQ-CSS score >95 points at enrolment* | 36 (18.5)  *8 (22.2)* | 13.28  *1.79* | 24.63  *7.92* |
| Decrease <5 points | 16 (8.2) | 4.76 | 12.98 |
| No change (score remains the same as at enrolment)  *Baseline KCCQ-CSS score >95 points at enrolment* | 14 (7.2)  *12 (85.7)* | 3.98  *3.22* | 11.75  *10.50* |
| **Total symptom score (TSS) (N=206)** |  |  |  |
| **Improvement (increase of ≥5 points as compared to enrolment)** | 81 (39.3) | 32.60 | 46.35 |
| Deterioration (decrease of ≥5 points as compared to enrolment) | 13 (6.3) | 3.40 | 10.55 |
| Stable (change of <5 points in either direction compared to enrolment) | 112 (54.4) | 47.30 | 61.31 |
| Increase <5 points  *Baseline KCCQ-TSS score >90 points at enrolment* | 50 (24.3)  *22 (44.0)* | 18.58  *6.81* | 30.72  *15.72* |
| Decrease <5 points | 31 (15.0) | 10.46 | 20.68 |
| No change (score remains the same as at enrolment)  *Baseline KCCQ-TSS score >90 points at enrolment* | 31 (15.0)  *25 (80.6)* | 10.46  *8.01* | 20.68  *17.39* |
| **Physical limitation score (PLS) (N=195)** |  |  |  |
| **Improvement (increase of ≥5 points as compared to enrolment)** | **78 (40.0)** | **33.07** | **47.24** |
| Deterioration (decrease of ≥5 points as compared to enrolment) | 12 (6.2) | 3.22 | 10.50 |
| Stable (change of <5 points in either direction compared to enrolment) | 105 (53.8) | 46.58 | 60.99 |
| Increase <5 points  *Baseline KCCQ-PLS score >90 points at enrolment* | 48 (24.6)  *22 (45.8)* | 18.74  *7.21* | 31.28  *16.58* |
| Decrease <5 points | 25 (12.8) | 8.47 | 18.34 |
| No change (score remains the same as at enrolment)  *Baseline KCCQ-PLS score >90 points at enrolment* | 32 (16.4)  *21 (65.6)* | 11.50  *6.79* | 22.37  *15.99* |

**Supplementary Table 5:** Proportion of patients with clinically relevant change in KCCQ-23 health status from enrolment at 12 months post-index among patients with paired enrolment and 12-month data

| **Association Between KCCQ-23 Improvement and Baseline Characteristics** | | | | |
| --- | --- | --- | --- | --- |
| Variable | Effect | Odds Ratio (OR) | 95% CI | p-value |
| **KCCQ-23 OSS Univariable Logistic Regression Results** | | | | |
| HF diagnosis < 65 years | Higher odds | 2.01 | 1.02-3.95 | 0.043 |
| History of MI | Lower odds | 0.51 | 0.26–0.97 | 0.041 |
| **KCCQ-23 OSS Multivariable Logistic Regression Results** | | | | |
| History of MI | Lower odds | 0.45 | 0.23-0.89 | 0.022 |
| **KCCQ-23 CSS Univariable Logistic Regression Results** | | | | |
| History of MI | Lower odds | 0.49 | 0.27-0.89 | 0.019 |
| **KCCQ-23 CSS Multivariable Logistic Regression Results** | | | | |
| HF hospitalization the prior 12 months | Lower odds | 0.47 | 0.23-0.95 | 0.036 |
| History of MI | Lower odds | 0.37 | 0.18-0.75 | 0.006 |
| **KCCQ-23 TSS Univariable Logistic Regression Results** | | | | |
| Ischemic origin of HF | Lower odds | 0.51 | 0.29–0.91 | 0.024 |
| HF duration ≤3 months | Lower odds | 0.57 | 0.34–0.99 | 0.044 |
| Presence of CAD | Lower odds | 0.57 | 0.33–0.98 | 0.042 |
| History of MI | Lower odds | 0.39 | 0.21–0.70 | 0.002 |
| Use of ICD and/or CRT | Higher odds | 2.81 | 1.38–5.69 | 0.004 |
| **KCCQ-23 TSS Multivariable Logistic Regression Results** | | | | |
| History of MI | 64 % Lower odds | 0.36 | 0.20-0.76 | 0.001 |
| Device therapy | Higher odds | 3.01 | 1.45-6.24 | 0.003 |
| **KCCQ-23 PLS Univariable Logistic Regression Results** | | | | |
| History of MI | Lower odds | 0.46 | 0.25-0.83 | 0.010 |
| **KCCQ-23 PLS Multivariable Logistic Regression Results** | | | | |
| History of MI | Lower odds | 0.47 | 0.25-0.85 | 0.013 |

**Supplementary Table 6:** Association Between KCCQ-23 Improvement and Baseline Characteristics

| **Multivariable logistic regression analysis of baseline characteristics associated with clinically relevant improvement in KCCQ-23 Scores from enrolment at any time over the study observation period** | | | | | |
| --- | --- | --- | --- | --- | --- |
|  | | | **95% CI** | |  |
| **Parameter** | **Category vs. reference** | **OR** | **Lower limit** | **Upper limit** | **p-value** |
| **KCCQ-23 Overall Summary Score; Number of observations used: N= 194** | | | | | |
| **Smoking status** | <20 vs ≥20 pack-years of smoking | 0.53 | 0.25 | 1.15 | 0.110 |
| **Patient’s age at initial HF diagnosis** | <65 vs ≥65 years | 1.88 | 0.94 | 3.74 | 0.074 |
| **Prior MI** | Yes vs No | 0.45 | 0.23 | 0.89 | 0.022 |
| **KCCQ-23 Clinical Summary Score; Number of observations used: N=190** | | | | | |
| **Smoking status** | <20 vs ≥20 pack-years of smoking | 0.53 | 0.24 | 1.17 | 0.116 |
| **Duration of HF at index date** | Continuous (in months) | 1.01 | 1.00 | 1.02 | 0.060 |
| **NYHA Class** | Class II vs III/IV | 0.51 | 0.26 | 1.01 | 0.055 |
| **Hospitalization for HF in the 12 months prior to index date (including the**  **hospitalization during which dapagliflozin was initiated, as applicable)** | Yes vs No | 0.47 | 0.23 | 0.95 | 0.036 |
| Prior MI | Yes vs No | 0.37 | 0.18 | 0.75 | 0.006 |
| **KCCQ-23 Total Symptom Score; Number of observations used: N= 217** | | | | | |
| Prior MI | Yes vs No | 0.36 | 0.20 | 0.67 | 0.001 |
| **Use of medical device (ICD and/or CRT)** | Yes vs No | 3.01 | 1.45 | 6.24 | 0.003 |
| **KCCQ-23 Physical Limitation Score; Number of observations used: N= 203** | | | | | |
| **Patient’s age at initial HF diagnosis** | <75 vs ≥75 years | 0.61 | 0.32 | 1.18 | 0.141 |
| **Hospitalization for HF in the 12 months prior to index date (including the**  **hospitalization during which dapagliflozin was initiated, as applicable)** | Yes vs No | 0.64 | 0.36 | 1.14 | 0.131 |
| Prior MI | Yes vs No | 0.47 | 0.25 | 0.85 | 0.013 |

**Supplementary Table 7:** Multivariable logistic regression analysis for the association of factors of interest with clinically relevant improvement in KCCQ-23 Scores from enrolment at any time over sthe study observation period

|  | **npt (%)** | **95% Clopper-Pearson CI** | |
| --- | --- | --- | --- |
|  |  | **Lower limit** | **Upper limit** |
| **Overall summary score (OSS) (N=176)** | | | |
| **Improvement (increase of ≥5 points as compared to enrolment)** | **104 (59.1)** | **51.44** | **66.43** |
| Deterioration (decrease of ≥5 points as compared to enrolment) | 22 (12.5) | 8.00 | 18.31 |
| Stable (change of <5 points in either direction compared to enrolment) | 50 (28.4) | 21.88 | 35.68 |
| Increase <5 points | 32 (18.2) | 12.78 | 24.69 |
| *Baseline KCCQ-OSS score >95 points at enrolment* | 5 (15.6) | 0.93 | 6.50 |
| Decrease <5 points | 12 (6.8) | 3.57 | 11.61 |
| No change (score remains the same as at enrolment) | 6 (3.4) | 1.26 | 7.27 |
| *Baseline KCCQ-OSS score >95 points at enrolment* | 5 (83.3) | 0.93 | 6.50 |
| **Clinical summary score (CSS) (N=195)** | | | |
| **Improvement (increase of ≥5 points as compared to enrolment)** | **106 (54.4)** | **47.09** | **61.49** |
| Deterioration (decrease of ≥5 points as compared to enrolment) | 23 (11.8) | 7.63 | 17.17 |
| Stable (change of <5 points in either direction compared to enrolment) | 66 (33.8) | 27.24 | 40.95 |
| Increase <5 points | 36 (18.5) | 13.28 | 24.63 |
| *Baseline KCCQ-OSS score >95 points at enrolment* | 8 (22.2) | 1.79 | 7.92 |
| Decrease <5 points | 16 (8.2) | 4.76 | 12.98 |
| No change (score remains the same as at enrolment) | 14 (7.2) | 3.98 | 11.75 |
| *Baseline KCCQ-OSS score >95 points at enrolment* | 12 (85.7) | 3.22 | 10.50 |
| **Total symptom score (TSS) (N=206)** | | | |
| **Improvement (increase of ≥10 points as compared to enrolment)** | **81 (39.3)** | **32.60** | **46.35** |
| Deterioration (decrease of ≥10 points as compared to enrolment) | 13 (6.3) | 3.40 | 10.55 |
| Stable (change of <10 points in either direction compared to enrolment) | 112 (54.4) | 47.30 | 61.31 |
| Increase <10 points | 50 (24.3) | 18.58 | 30.72 |
| *Baseline KCCQ-OSS score >90 points at enrolment* | 22 (44.0) | 6.81 | 15.72 |
| Decrease <10 points | 31 (15.0) | 10.46 | 20.68 |
| No change (score remains the same as at enrolment) | 31 (15.0) | 10.46 | 20.68 |
| *Baseline KCCQ-OSS score >90 points at enrolment* | 25 (80.6) | 8.01 | 17.39 |
| **Physical limitation score (PLS) (N=195)** | | | |
| **Improvement (increase of ≥10 points as compared to enrolment)** | **78 (40.0)** | **33.07** | **47.24** |
| Deterioration (decrease of ≥10 points as compared to enrolment) | 12 (6.2) | 3.22 | 10.50 |
| Stable (change of <10 points in either direction compared to enrolment) | 105 (53.8) | 46.58 | 60.99 |
| Increase <10 points | 48 (24.6) | 18.74 | 31.28 |
| *Baseline KCCQ-OSS score >90 points at enrolment* | 22 (45.8) | 7.21 | 16.58 |
| Decrease <10 points | 25 (12.8) | 8.47 | 18.34 |
| No change (score remains the same as at enrolment) | 32 (16.4) | 11.50 | 22.37 |
| *Baseline KCCQ-OSS score >90 points at enrolment* | 21 (65.6) | 6.79 | 15.99 |

**Supplementary Table 8:** Proportion of patients with clinically relevant change in KCCQ-23 health status from enrolment at 12 months post-index among patients with paired enrolment and 12-month data
